# The relationship between intestinal goblet cells and the immune response

**DOI:** 10.1042/BSR20201471

**Published:** 2020-10-16

**Authors:** Mingming Zhang, Chenchen Wu

**Affiliations:** College of Animal Veterinary Medicine, Northwest A & F University, Yangling 712100, Shaanxi, People’s Republic of China

**Keywords:** Goblet cells, Immune response, Mucins

## Abstract

Goblet cells (GCs) are single-cell glands that produce and secrete mucin. Mucin forms a mucus layer, which can separate the materials in cavities from the intestinal epithelium and prevent the invasion of pathogenic microorganisms in various ways. GCs can also participate in the immune response through nonspecific endocytosis and goblet cell-associated antigen passages (GAPs). GCs endocytose soluble substances from the lumen and transmit antigens to the underlying antigen-presenting cells (APCs). A variety of immuno-regulatory factors can promote the differentiation, maturation of GCs, and the secretion of mucin. The mucin secreted by GCs forms a mucus layer, which plays an important role in resisting the invasion of foreign bacteria and intestinal inherent microorganisms, regulating the immune performance of the body. Therefore, the present study mainly reviews the barrier function of the mucus layer, the mucus secreted by goblet cells, the protective effect against pathogenic bacteria, the delivery of luminal substances through GAPs and the relationship between GCs and the immune response.

## Introduction

The intestine is one of the important digestive organs of the human and animal body, but there are large numbers of bacteria, viruses and parasites in the intestine, which is a potential source of infection [1]. In general, intestinal mucosal barriers include mechanical, immune, and biological barriers [2]. These barriers play a significant role in maintaining the intestinal micro-ecological balance and the stability of the internal environment, preventing the invasion of pathogens and the displacement of endotoxins, and regulating the microbial–host immune response [3]. The mucus on the surface of the intestinal mucosa participates in forming a mucus barrier and plays an important role in protecting the epithelium [4]. Goblet cells (GCs) are formed by the differentiation of intestinal epithelial cells. GCs are single-cell glands whose main function is to synthesize and secrete mucins. Mucin 2 (MUC2), water, and inorganic salts constitute the mucus layer, in which water accounts for more than 90%; thus, the layer is colloidal. The mucus layers in the small and large intestines are different. In the small intestine, the mucus and antibacterial peptides/proteins form an antibacterial gradient, reducing the number of bacteria that can reach the epithelial cells. In the colon, normally, bacteria cannot penetrate its two mucus layers. The penetration of the inner layer by bacteria can lead to inflammation [5,6]. Mucin can prevent the loss of sIgA antibody molecules on the intestinal cavity side of epithelial cells because sIgA can interact with mucin and bacterial cell surface proteins to stabilize the bacterial biofilm [7]. sIgA in the mucus layer will be phagocytosed and cleared by macrophages after binding to bacteria or antigens.

Therefore, the main content of this review includes the defense function of the mucus layer and sIgA, the classification and function of GCs, the secretory function of mucin, the regulatory pathway of immune factors on mucin secretion, and the mechanism of immune factors on GCs and mucin. The introduction of the above content will lay a theoretical foundation for further study of the relationship between GCs and the immune response.

## The histomorphology and distribution of goblet cells

GCs are single-cell glands that exist in humans and mammals and are present in the epithelium, including the respiratory, digestive tracts, and genital atrium. GCs are present in the intestinal epithelial cells in the early postnatal period [8]. As a highly polarized columnar epithelial cell, the tops of GCs are enlarged, and the bottom of the cytoplasm is narrow and located on the basement membrane. The nucleus is small, located at the base, triangular or oblate, and stained deeply. GC has a large perinuclear region with the endoplasmic reticulum, Golgi apparatus, and concentrated vesicles. The pluripotent stem cells at the base of the intestinal crypt differentiate to form GCs [9]. The cytoplasm is filled with thick secretory granules containing mucin, a glycoprotein. GCs are known for their secretion of mucin, providing the mucosal surfaces with a thick mucus lining that acts as a barrier to limit interactions with microbes. The migration and differentiation to the shedding of GCs occur over a total of 2–4 days [10]. The distribution of GCs in the intestinal epithelium increases gradually from the duodenum to the distal colon [11], from 4% to 16%, consistent with the increase in the distribution of the intestinal flora.

## The defense function of the mucus layer

### Protective effect of the mucosal layer on the intestinal epithelium

There are a large number of microorganisms in the intestine. The normal human gut microbiota comprises two major phyla, namely Bacteroidetes and Firmicutes [12]. The intestinal microflora is divided into physiological bacteria, conditioned pathogenic bacteria and pathogenic bacteria [13]. These bacteria may cause damage to the body through the mucosal barrier, making the intestine a potential source of infection [14]. The relationship between intestinal bacteria and the mucosal barrier is well-balanced in the stable state; intestinal bacteria cannot contact the intestinal epithelium. However, when the mucosal barrier is dysfunctional, intestinal bacteria can approach the intestinal immune cells and cause inflammatory bowel disease (IBD) [15]. Mucus serves as a semi-permeable gel layer, which allows the exchange of gases, water and nutrients with the underlying epithelium [16]. The mucosal epithelium is characterized by a mucus layer on the surface, which plays an important role in separating the intestinal epithelium from the cavity material, resisting the invasion of exogenous bacteria and intestinal microorganisms and maintaining the intestinal micro-ecological balance.

The thickness of the gel-forming mucins formed by the secreted glycoproteins on the surfaces of intestinal epithelial cells ranges from 300 to 700 μm, preventing microorganisms from penetration. The transmembrane mucins, such as MUC1 and MUC3, MUC12, MUC13 and MUC17, cover the apical surfaces of the enterocytes at a thickness of 30–500 nm in a structure called glycocalyx, which can also play a physically protective role. The mucous layer is constantly updated and removes pathogenic substances from the intestinal lumen.

The mucus layer not only plays roles as a source of lubrication [17] and a physical barrier but also captures microorganisms and acts as a trap for microbes. The mucus provides a matrix for a rich array of antimicrobial molecules [18]; mucin oligosaccharides can bind to microorganisms, and the structures and negatively charged properties of glycoproteins are beneficial for encapsulating bacteria. The exposed chemical groups are similar to the surface structure of the intestinal epithelium, making it easy for bacteria to recognize and attach. Mucin oligosaccharides have an antibacterial activity or can carry other antibacterial molecules in some cases. Mucins can bind antibacterial molecules such as histatins and statherin to make them better protect the host in the correct mucosal microenvironment [19]. Experiments have shown that MUC2 binds to luminal antigens (especially bacteria) and reacts with dendritic cells (DCs) to suppress inflammatory [20].

**Figure 1:**
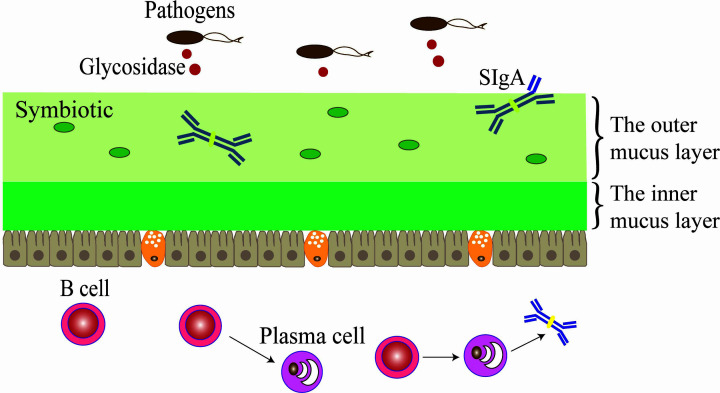
Regulatory mechanism of sIgA on the barrier function of the colon mucus layer The colon has two layers of mucus layers. The inner mucous layer of the colon is tightly attached to the epithelial cells and cannot be infiltrated by bacteria, and the outer mucus layer is relatively loose; symbiotic bacteria and sIgA are colonized in the outer mucus layer.

The colon has two layers of mucus, while the small intestine has one [21]. The loose outer mucus layer of the colon is the habitat of commensal bacteria. The relatively dense inner mucus layer is firmly attached to the epithelial cells and the concentration of MUC2 is higher. The inner mucus layer is impervious to bacteria [22] and is renewed every hour by surface GCs [23] (Figure 1). The mucus of the small intestine and antibacterial peptides function together to form an antibacterial mucus gradient, preventing bacteria from reaching epithelial cells.

Microbes, microbial products and cytokines regulate the production and secretion of mucus [24]. Microbiota affects the composition and thickness of mucus. Germ-free mice have fewer GCs and a thinner mucus layer than conventionally raised mice [25,26]. PH affects the rheological property of mucus [27,28]. *Helicobacter pylori* can invade the mucus barrier by increasing the pH in the environment and reducing the viscoelasticity of mucin [29]. Parasympathetic drugs promote mucus secretion of crypt goblet cells [30]. Immunomodulatory prostaglandins (cAMP-mediated agonists) and carbachol (Ca^2+^-mediated agonists) produce significant mucus release [31]. The prostaglandin stimulation rate can reach about 350 pl/min, which is a long-lasting stimulus [32].

Under normal circumstances, pathogenic bacteria rarely colonize the intestinal lumen; however, when the intestinal flora is displaced or the flora is out of balance, a large number of pathogenic bacteria can colonize and expand, causing disease. The bacteria’s exoglycosidase breaks down the polysaccharides in the mucin, and the mucin layer dissolves when it touches the core of the protein [33]. The depletion of GCs leads to defects in the mucus layer, increased adhesion of bacteria on the surface of the epithelium, and reduced digestion and absorption of nutrients [34,35]. Mucin changes and the dysfunction of the mucosal barrier is related to the occurrence and development of IBD such as ulcerative colitis (UC) and Crohn’s disease (CD) [36,37].

### Mucin and sIgA contribute to the barrier function

Secretory immunoglobulin A (sIgA) is the main immunoglobulin on the intestinal mucosal surface and plays an important role in preventing the attachment and colonization of pathogenic bacteria and protecting the gastrointestinal mucosa [38,39]. sIgA is secreted into the intestinal cavity and is mixed in the mucous layer, which covers the surface of epithelial cells and binds microorganisms or food antigens to form antigen–antibody complexes, facilitates phagocytosis and removal of macrophages. sIgA cannot bind to mucus in the absence of carbohydrates and cannot stop infections caused by bacteria in a murine respiratory infection model [40]. It has been proven that sIgA–mucin protein interaction is an important factor for sIgA to capture pathogens.

Immunofluorescence microscopy determined that sIgA co-located with intestinal bacteria in the outer mucous layers of mouse and human colon. It was demonstrated that sIgA anchored the outer mucous layer by acting in conjunction with mucinous proteins and intestinal bacteria [41], thus playing a role in the immune defense to germs and maintaining a mutually beneficial symbiosis with symbiotic bacteria, protecting epithelial cells. Using pIgR and the mucin-2 deficient mice demonstrated that Muc2 is needed instead of sIgA for bacteria excreting from the inner mucus layer of the colon [42]. The above points indicated that sIgA promotes mucosal colonization of microbiota with beneficial properties, whereas disease states may induce sIgA responses to pathogens that disrupt the equilibrium of the healthy microbiome.

## The mucus of secretory goblet cells

As secretory intestinal epithelial cells, GCs function to synthesize and secrete mucin. Mucin is a high-molecular-weight glycoprotein, the synthesis process of which includes dimerization in the endoplasmic reticulum, Golgi glycosylation, and finally oligomerization [43]. It is stored in particles in the GCs, and after being released, it forms a network structure on the surface of the intestinal epithelium. The major mucin in the intestine is MUC2, which is highly glycosylated mucin. Its protein skeleton binds to various O-link oligosaccharides [44].

Mucin genes are named MUC1 to MUC21 according to their sequence of discovery [45]. In terms of their structure and location, mucins can be divided into the secretory type and membrane-bound type. Secretory mucins include MUC2, MUC5AC, MUC5B, MUC6, MUC19 [46,47] (secreted gel-forming mucins) and MUC7 [48], MUC8 [49], MUC9 [50] (secreted non-gel-forming mucins). Membrane-bound types include MUC1, MUC3A, MUC3B, MUC4, MUC12, MUC13, MUC16, MUC17, and MUC20. According to their functions, they are divided into transmembrane mucin and secretory mucin. Structurally, transmembrane mucin includes a transmembrane domain in its C-terminus, which is involved in intracellular signal transduction. Different mucins are expressed at different sites *in vivo*: the stomach and duodenum can express MUC6; MUC5B is expressed in the human colon at low levels; and MUC5AC is expressed in the stomach and the intestine after infection [51].

All mucins contain a large number of serine (P), threonine (T) and proline (S) residues, called PTS domains. These residues are also highly glycosylated, and their N- and C-termini are rich in asparagine and cysteine. Secretory granules, which are formed by completely glycosylated mucins, are filled and stored in the GCs. The secretion including the constitutive and regulatory pathways, regulated by a variety of biological activity factors. The constitutive pathway continuously secretes mucin to maintain the renewal of the intestinal mucus layer, while the regulatory pathway functions in cases of pathological or environmental stimulation, such as bacterial infection and inflammation [52].

## Sentinel goblet cells and nonspecific endocytosis

Previous studies have shown that GCs secretion of mucin is associated with autophagy, NLRP6 (NOD-like receptor family pyrin domain-containing 6) and caspase 1/11 [53–55]. GCs secrete mucin granules in the colon exposed to ischemia–reperfusion, remove bacteria from the colonic crypts, and restore the mucus layer during reperfusion [56].

**Figure 2:**
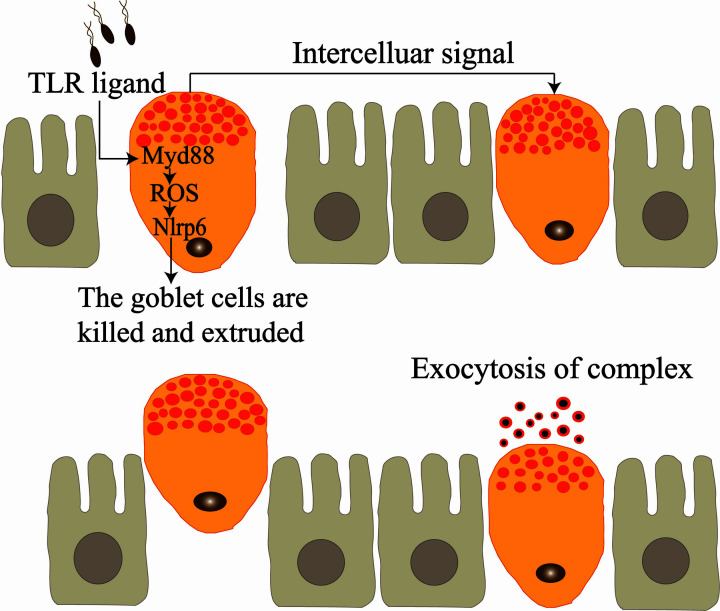
Regulatory mechanism of the goblet cell endocytosis signaling pathway After stimulating goblet cells, the bacterial TLR ligand activates the intracellular signaling pathway, which in turn activates Myd88, ROS synthesis, and Nlrp6, triggering the exocytosis of the complex and the secretion of MUC2 by adjacent goblet cells. The goblet cells that undergo non-specific endocytosis are killed and expelled to the lumen.

Stem cells at the bottom and middle of the intestinal crypts continue to differentiate to maintain the self-renewal of the intestinal epithelium [57]. Recently, it has been discovered that some GCs at the entrance of colonic crypts can undergo nonspecific endocytosis, called sentinel goblet cells (senGCs). The senGCs, in turn, activate the TLR (Toll-like receptor) signal and MyD88 (myeloid differentiation factor 88) dependent reactive oxygen species synthesis of Nox/Duox (NADPH oxidase/dual oxidase) and the NLRP6 inflammasome-mediated activation of caspases 1 and 11, thereby reacting with and endocytosing the ligands of TLR2/1, TLR4 and TLR5, causing the complex to trigger the exocytosis of Muc2 and intercellular signaling connections. Adjacent responsive GCs are induced to secrete MUC2, and the bacteria are washed out from the opening of the crypt, protecting it (Table 1). The endocytosed senGCs are killed after the complex exocytosis occurs and are discharged from the epithelium into the lumen, a process driven by the activation of the NLRP6 inflammasome (Figure 2) [58].

**Table 1:** Mucus production and immunomodulatory molecules and pathways

Trigger	Cell	Pathway	Result
**TLR ligand**	Sentinel goblet cells (senGCs)	TLR, MyD88, NLRP6	Exocytosis of Muc2 and adjacent responsive GCs are induced to secrete MUC2
**adverse conditions such as bacterial infection or antibioticS**	GCs in the small intestine and distal colon	Myd88, EGRF and p42/p44 MAPK	Inhibit the formation of GAPs

Mucin production is regulated by many factors, including temperature, pH value, infection, and inflammatory factors. When the body is threatened by bacterial infection, it will activate the GCs pathway and secrete mucus to prevent pathogens from approaching the intestinal epithelium.

GCs can rapidly deal with pathogens that may cause intestinal epithelial infection by infiltrating the mucosal barrier through non-specific endocytosis, secreting mucus to maintain the mucous layer without activating the adaptive immune system. Although GCs die in this process, they play an important role in protecting intestinal mucosa and intestinal epithelium [59].

## The immune signal mechanisms of Goblet cell-associated antigen passages

GCs in the small intestine and distal colon can form goblet cell-associated antigen passages (GAPs). GAPs are the main mechanism for delivering luminal small soluble antigens to intestine lamina propria (LP) DCs in a stable state [60–62]. GAPs are formed by GCs when acetylcholine (ACh) acts on muscarinic acetylcholine receptor 4 (mAChR_4_), antigen-presenting cells (APCs) migrate to the intestinal epithelium and acquire antigen in a mAChR_4_-dependent manner.

GAPs in the small intestine form 18 days after weaning. Colon GAPs are formed before weaning and are suppressed after weaning [63]. The formation of the proximal colon GAPs is inhibited by GC intrinsic microbial sensing. Colon microbes inhibit the formation of the GAPs in a myd88-dependent manner, Myd88 activates epidermal growth factor receptor (EGRF), and p42/p44 mitogen-activated protein kinase (MAPK), which makes them phosphorylated to inhibit the formation of colon GAPs (Table 1) [64]. The proximal colon has a higher density of bacteria than the small intestine [65], and a thinner mucus layer than the distal colon [66]. Through the inhibition of microbial sensing, the immune system of the proximal colon is prevented from being exposed to the bacteria in the cavity and the inflammatory reaction is avoided. Opening colonic GAPs for 4 days leads to increased levels of neutrophil chemokine CXCL1 and inflammatory factors IL-6, IL-17, as well as the influx of colonic neutrophils.

The antigen delivered by the GAPs in the small intestine is mainly delivered to CD103^+^ DCs, which can stimulate the proliferation of T cells and induce an adaptive immune response. Particles smaller than 20 nm can enter the small intestine GAPs. LP-DCs slowly collect or actively detect antigens. Some GCs in the small intestine form GAPs when they secrete, but not all GCs secretion is related to the formation of the GAPs. GAPs are also found in human jejunum [67]. The intestine LP-APCs include CD11b^+^CD103^−^CX_3_CR1^+^ APCs and CD11b^+^CD103^+^CX_3_CR1^−^ APCs [68], collectively known as mononuclear phagocytes (MNPs), both of which can react with GAPs in the small intestine and colon. The more frequent reaction between CD103^+^ LP-APCs and GAPs may be due to the stronger migration ability of CD103^+^ LP-APCs, the response ability to granulocytes and inflammatory factors, and the stimulation ability to T cells [69].

The use of antibiotics causes inflammation and damages the intestinal mucosa and epithelium. Antibiotics reduce the inhibition of the formation of colon GAPs, allowing commensal bacteria and protein antigens in the intestinal lumen to translocate across the intestinal epithelium through the GAPs [70]. LP-MNPs ingest commensal bacteria and protein antigens and migrate to the draining lymph nodes (LNs) to promote inflammatory response, leading to the response of inflammatory T cells to other innocuous antigens [71].

Salmonella can pass through the intestinal epithelium through GAPs in the early stage of infection [72]. IL-1β participates in the inhibition of GAP formation in the small intestine during Salmonella infection. Besides, Listeria monocytogenes has also been proved to pass through the intestinal epithelium through targeting GCs [73], indicating that the pathogenic bacteria may use GAPs as an invasion portal. It is speculated that under disadvantaged conditions such as bacterial infection, GCs may secrete mucus to maintain the mucus layer, inhibit the formation of GAPs, and thus prevent the immune system from being inappropriately exposed to intraluminal substances. GAP formation is highly adaptable to different luminal conditions [74].

GAPs are also related to oral tolerance [75]. Oral tolerance is the state in which the immune system has no immune response to innocuous antigens such as food antigens. Oral tolerance generates regulatory T cells (Tregs), which play an important role in maintaining immune homeostasis [76]. GAPs support the induction and maintenance of oral tolerance by delivering luminal antigens, maintaining pre-existing LP Tregs, and imprinting tolerogenic properties on LP-APCs [77].

## Effects of inflammatory factors on goblet cells and mucus secretion

### Effects of Th2 secreted cytokines on goblet cells and mucus secretion

Intestinal infection with parasites causes the immune response to skew toward type 2 helper (Th2) cells, leading to increased secretion of cytokines such as IL-4 [78], IL-5, IL-9, and IL-13. Among them, IL-13 plays a dominant role and a vital cytokine regulating GC proliferation. IL-13 can act on epithelial cells by acting on signal transducer and activator of transcription-6 (STAT6) and promotes GC proliferation. Experiments have shown that GC proliferation was detected in IL-13 overexpressing mice, and the overexpression of exogenous IL-25 and IL-9 also promoted GC proliferation and mucin expression through an IL-13-dependent pathway [79]. In the LP of the small intestine, IL-13 plays a role in expelling worm through GCs hyperplasia [80]. IL-4 and IL-13 up-regulate intestinal trefoil factor (ITF) and MUC2 transcription in human colon cancer cell lines [81,82]. GCs proliferation caused by helminth infection is mainly controlled by IL-4 and IL-13 in the Th2 immune response, but the GCs proliferation caused by Syphacia obvelata and Schistosoma mansoni infection is independent of IL-4 and IL-13 [83].

Th2 cytokines, including IL-4, 1L-6, IL-9, 1L-10, and IL-13 [84]. IL-9 and IL-10 have also been shown to regulate mucin expression. Interleukin-9 up-regulates mucus expression in the airways [85]. IL-10 promotes the production of intestinal mucus by suppressing protein misfolding and endoplasmic reticulum stress in GCs [86].

**Figure 3:**
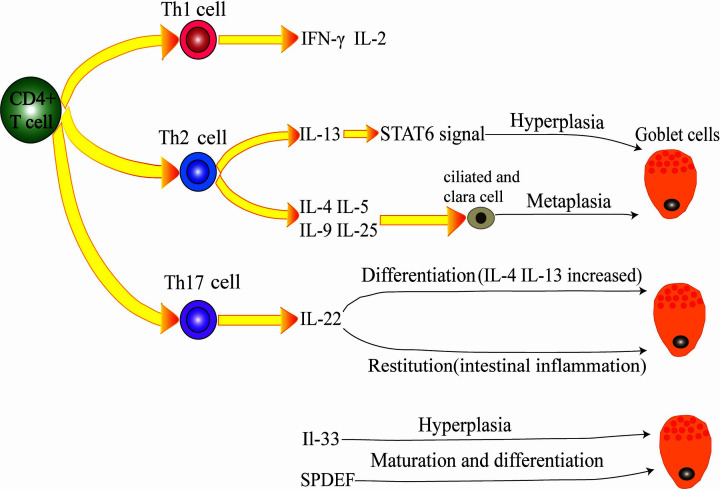
Inflammatory factors regulate goblet cell secretion CD4 Th cells include Th1, Th2, and Th17 cells. Th2 cells secrete IL-13, which can promote goblet cell proliferation through STAT6 signaling. Th2 cells can also secrete IL-4, IL-5, IL-9, and IL-25 to promote the metaplasia of ciliated cells and Clara cells into goblet cells. Th17-secreted IL-22 can regulate goblet cell differentiation and promote goblet cells in the case of intestinal inflammation. Other cytokines, such as IL-33 and SPDEF, can also affect goblet cells. IL-33 promotes the proliferation of goblet cells, and SPDEF regulates the maturation and differentiation of goblet cells.

Th2 cells promote not only the proliferation of GCs but also the metaplasia of ciliated and Clara cells into GCs by releasing IL-4, IL-5, IL-9 and IL-25, followed by increased mucus secretion and airway epithelial thickening [87,88] (Figure 3).

### Effects of Th17 secreted cytokines on goblet cells and mucus secretion

Th17-related IL-22 can regulate GC differentiation and mucin expression. IL-22^−/−^ mice had lower levels of mucin expression and GC numbers in response to Nippostrongylus brasiliensis and Trichuris muris infections compared with wild-type mice and increased contents of IL-4 and IL-13 [89]. During intestinal inflammation, IL-22 can promote MUC1 levels and the restitution of GCs, increasing the mucus barrier and epithelial regeneration. This process is mediated by Th1 or Th2 [90]. There may be overlap in the process by which inflammatory factors promote GC differentiation and mucin expression. IL-22 gene delivery leads to increased STAT3 (signal transducer and activator of transcription-3)-dependent mucin secretion and GCs repair, rapidly alleviate local intestinal inflammation [91].

A typical cytokine, IL-17A, is also released by Th17 cells. IL-17A can induce the release of many cytokines, and TNF-ɑ, IL-1β, G-CSF, IL-6, CXCL2 and IL-8, IL-17A and IL-6 together can promote the expression of MUC5AC and MUC5B [92]. Both Th2 and Th17 cells exhibit significantly increased expression of the MUC5AC gene in airway epithelial cells *in vitro and in vivo* [93].

### Effects of other cytokines on goblet cells and mucus secretion

IL-33 promotes GC proliferation and mucin expression. IL-33 induce MUC5AC expression, and also GC hyperplasia at air–liquid interface culture in human nasal epithelial cells [94]. IL-33 does not directly act on epithelial cells but indirectly induces GCs differentiation through IL-13 produced by group 2 innate lymphoid cells [95]. The transcription factor SPDEF (SAM pointed domain ETS factor) can regulate GCs in multiple organs. SPDEF regulates the proliferation of GCs in the airway, which is related to its maturation; SPDEF is also related to the maturation and differentiation of GCs in the small intestine and plays a role in promoting maturation and differentiation of GCs in the lung [96,97].

## Conclusion

The mucus layer prevents microorganisms from approaching the gastrointestinal epithelial cells and moves continuously to remove residual material. Mucus can capture pathogenic bacteria and can also serve as a matrix for antibacterial substances. Thus, the pathogen needs a unique pathway to break into the mucin and invade the mucous layer. The dynamic properties of the intestinal mucus layer and its regulation of the intestinal microflora and local immune system are very complex. The different distributions of the mucous layer in the intestinal lumen and the pleiotropic effect of mucin during inflammation suggest that the intestinal mucous barrier is not only an independent component but also closely related to the intestinal mucosal physics and immune barrier. The temporal and spatial distributions of the bacteria in mucus and their interaction with the local immune system remain challenges for future research. Our research group is interested in this research work and hopes to provide strong evidence for the function of the mucus layer barrier.

## Abbreviations

ACh: acetylcholine
APC: antigen-presenting cell
CD: Crohn’s disease
DC: dendritic cell
EGRF: epidermal growth factor receptor
GAP: goblet cell-associated antigen passages
GC: Goblet cell
IBD: inflammatory bowel disease
ITF: intestinal trefoil factor
LN: lymph nodes
LP: lamina propria
mAChR_4_: muscarinic acetylcholine receptor 4
MAPK: mitogen-activated protein kinase
MUC2: mucin 2
MNP: mononuclear phagocytes
NLRP6: NOD-like receptor family pyrin domain-containing 6
Nox/Duox: NADPH oxidase/dual oxidase
senGC: sentinel goblet cell
sIgA: secretory immunoglobulin A
SPDEF: SAM pointed domain ETS factor
STAT3: signal transducer and activator of transcription-3
STAT6: signal transducer and activator of transcription-6
Th2: type 2 helper
TLR: Toll-like receptor
Treg: regulatory T cell
UC: ulcerative colitis
